# Preclinical Studies of Posttraumatic Headache and the Potential Therapeutics

**DOI:** 10.3390/cells12010155

**Published:** 2022-12-30

**Authors:** Mikiei Tanaka, Yumin Zhang

**Affiliations:** Department of Anatomy, Physiology and Genetics, Uniformed Services University of Health Sciences, 4301 Jones Bridge Rd, Bethesda, MD 20814, USA

**Keywords:** posttraumatic headache, traumatic brain injury, trigeminovascular system, CGRP, endocannabinoids, migraine

## Abstract

Posttraumatic headache (PTH) attributed to traumatic brain injury (TBI) is a secondary headache developed within 7 days after head injury, and in a substantial number of patients PTH becomes chronic and lasts for more than 3 months. Current medications are almost entirely relied on the treatment of primary headache such as migraine, due to its migraine-like phenotype and the limited understanding on the PTH pathogenic mechanisms. To this end, increasing preclinical studies have been conducted in the last decade. We focus in this review on the trigeminovascular system from the animal studies since it provides the primary nociceptive sensory afferents innervating the head and face region, and the pathological changes in the trigeminal pathway are thought to play a key role in the development of PTH. In addition to the pathologies, PTH-like behaviors induced by TBI and further exacerbated by nitroglycerin, a general headache inducer through vasodilation are reviewed. We will overview the current pharmacotherapies including calcitonin gene-related peptide (CGRP) monoclonal antibody and sumatriptan in the PTH animal models. Given that modulation of the endocannabinoid (eCB) system has been well-documented in the treatment of migraine and TBI, the therapeutic potential of eCB in PTH will also be discussed.

## 1. Introduction

Traumatic brain injury (TBI) is caused by external mechanical forces such as compressive, tensile, and shear forces to the head resulting in mortality and long-term disability. More than 60 million people worldwide are affected by TBI each year and about half of the world’s population have one or more TBI over their lifetime [1,2]. Human TBI cases show that TBI is not a simple pathophysiological event, but exhibits heterogeneous and complex pathologies and symptoms depending on the types of impacts and other factors such as age and sex. One of the most common sequelae of TBI is acute or chronic headache that is known as posttraumatic headache (PTH) and is commonly seen in patients with mild TBI (mTBI). According to the International Classification of Headache Disorders, PTH is defined as a secondary headache occurring within 7 days following trauma or after recovery from unconsciousness [3]. Most cases are acute PTH that resolves within 3 months, but in a substantial number of patients, PTH can become persistent and last for more than 3 months, and even up to several years [4]. PTH patients either with migraine-type symptom experiencing pulsating pain or with tension-type headache experiencing bilateral pressing pain often report nausea, photophobia and phonophobia [5,6]. TBI patients with acute and chronic PTH also suffer from anxiety and depression that are more commonly seen than those without PTH [7,8]. In general, PTH has clinically similar symptoms to migraine or tension-type headache, and therefore many PTH patients currently receive the primary headache therapies and preventive treatments. However, there are scarce evidence-based approaches and the overall pain management does not meet satisfaction [9,10], due to the poor understanding on the PTH pathogenic mechanisms. Although no PTH animal models can imitate the complex clinical conditions, they do provide valuable information and insights on the cellular and molecular mechanisms that are difficult to achieve without preclinical animal modeling. Furthermore, preclinical studies are indispensable for the development of therapeutics. In this narrative review article, we mainly focus on the preclinical studies using the animal models of TBI and discuss the pathological features in the trigeminal system attributing to the PTH pathogenesis. The pharmacotherapies including anti-CGRP therapy and endocannabinoids (eCBs) system modulation as potential PTH treatments will also be discussed.

## 2. Posttraumatic Headache Animal Models

### 2.1. TBI Induced PTH Models

It is known that brain tissue damage by TBI occurs in two-step processes: Primary and secondary injuries. During the primary injury, brain tissue deformation occurs and neurons can undergo disruptive morphological changes and cell death in the impact areas by mechanical force or penetration. From minutes to months after primary injury, secondary injury evolves as the results of consequent metabolic, cellular and molecular events which spread the injury to the surrounding and distant areas. There are several TBI models that aimed to replicate the pathological and symptomatic characteristics of human patients. TBI models are basically classified into two major types either with a focal or a more diffuse injury. In the focal injury models, impact is directly impacted to the brain such as the sensorimotor cortex, where the most pathological damages are observed. Accordingly, brain damage is relatively limited to the regions of impact. The typical focal injury model is induced by the controlled cortical impact, which utilizes a pneumatic piston to impact on the desired brain areas [11]. In the diffuse injury model, animals are given the impact to trigger a rapid head movement. The rotational acceleration and deceleration of head generates stretching and shearing force on axon fibers in dispersed brain regions, and the damage occurs not only on axonal fibers but also on microvasculature. A commonly used diffuse injury model is induced by blast exposure in the shock tube, in which a supersonic blast wave impulse was exposed to animals [12]. Some models provide both focal and diffuse injuries, for instance, the lateral or midline fluid percussion model, in which fluid pressure hits directly to the exposed dura through the implanted Lure-Lock [13,14]. The weight-drop injury model uses gravitational free-falling weight onto a positioned brain skull to generate concussive impact [15,16]. This injury model was later modified by dropping the weight on the head placed on a soft platform, which allows rapid head movement to cause rotational acceleration and deceleration [17]. In addition to the injury type, the strength of impact and its frequency are the major variables impacting the disease outcomes. For instance, TBI induced by a repeated weight drop showed a differential extracephalic allodynia duration pattern and different response to CGRP mAb treatment compared to that by a single heavy weight drop [18]. It is also important to elucidate the pathogenic mechanisms of repetitive TBI, since the professional sports athletes and military personnel often suffer from repetitive TBI and may have sustained PTH. To date, lateral fluid percussion model, controlled cortical impact model, weight drop model, and blast exposure model have all been used for PTH studies. In each of these model systems, the injured animal exhibits increased periorbital allodynia and headache-related behavioral changes as described in the following sections. However, the underlying mechanisms attributing to PTH-like behavior and pathophysiology may differ in the different TBI model systems [19,20].

### 2.2. Behavioral Characterization in the PTH Models

#### 2.2.1. Cephalic and Extracephalic Allodynia

As animals cannot verbally express pain, the pain-related behavioral changes are commonly used as headache indicators. Allodynia is one of the characteristics of headache sufferers including patients with head trauma [21,22], which supports the relevance to pathophysiology in the human cases. Thus, cranial mechanical hypersensitivity measured by von Frey test has been widely used in the PTH study, and the facial expression assay based on the Rat Grimace Scale is also utilized in the rodent inflammatory and neuropathic pain models [23,24]. By applying von Frey filaments on the surface of the periorbital area, the tactile sensitivity is assessed based on the animal’s response such as head shaking and scratching the touched areas. The tactile sensitivity threshold is mostly determined by the up-down method using multiple von Frey monofilaments [25,26]. Before starting the test, animals are well-adapted to the experimental conditions to minimize the stress. The hind paw tactile sensitivity is often measured, because central sensitization may result in both peripheral and central pain [27].

A number of studies have shown cephalic hyperalgesia in TBI animal models, though the duration is varied ranging from days to months post-injury depending on the severity and type of injury. For instance, Wattiez et al. examined allodynic behavior in two different TBI models; severe compressive impulse induced variable shock front and acceleration/deceleration insult caused hypersensitivity in both cephalic and extracephalic regions for at least 63 days, whereas the repeated weight drop model resulted in cephalic allodynia for 2 weeks without altering plantar tactile pain threshold [20]. The sustainability seems to be sex dependent [28]. In addition to cephalic algesia, several studies also examined extracephalic algesia mainly by von Frey test in the hind paw. There was no alteration on the hind paw tactile sensitivity in the weight drop model irrespective of the cephalic hyperalgesia [20,29]. However, other studies have reported that extracephalic hyperalgesia from 24 h to several weeks post-injury was similar to cephalic hyperalgesia observed in the weight drop model [30], as well as in the lateral fluid percussion model [31]. Overall, extracephalic algesia is less consistently observed than cephalic algesia in the PTH models, suggesting that TBI-induced pain mechanisms between cephalic and extracephalic regions are overlapped to some extent, but are also independently regulated. A method for assessment of orofacial pain sensitivity has been developed based on the conflicting behavior of reward intake under the thermal aversive environment [32]. Using this method, rats with mild closed head injury showed an increased thermal sensitivity to both hot (42 °C) and cold (22 °C) temperature, although there was no significant difference under the innoxious (37 °C) temperature [33]. On the other hand, Uddin et al. found that the blast injury animals showed less reward consumption at 7 °C, but not at hot temperature [34]. Consistently, the Rat Grimace Scale was significantly elevated in the blast injury rat model compared to that in the sham animals [35]. Although the statistical significance of this assessment was smaller than that reported by the von Frey test, orofacial allodynia might be positively correlated with the painful facial expression examined by Rat Grimace Scale score [35]. In a polytrauma model induced by both limb fracture and TBI, female animals showed prolonged hind paw allodynia compared to male animals [36], which may help to explain the sex differences in the human PTH cases.

#### 2.2.2. Photophobia

Photophobia, a sensory disturbance provoked by light, is the main symptom of migraine sufferers as well as the PTH patients [37]. Consistent with the human cases, photophobia-like behavior was also reported in the TBI animal model induced by controlled cortical impact [38]. Animals were tested using the Light/Dark box apparatus with the light area under ambient or bright light conditions. Although both sham and TBI animals spent similar time period in the light area under ambient light conditions, the TBI animals spent less time in the light area than control animals under bright light conditions. In addition, under the bright light conditions, PTH animals had significantly reduced rearing behavior compared to the sham control animals, suggesting that the exploratory behavior in the brightness was affected in the injured animals. In the weight drop model, exposure to the bright illumination reinstated cutaneous periorbital and hind paw allodynia 2 weeks post-injury even after cutaneous allodynia induced by TBI was resolved on day 13 post-injury [30]. This phenomenon was clinically relevant, since reduction of heat pain threshold immediately after bright light exposure was reported in PTH patients [37]. Despite only a few reports address the photophobia-like behavior in the animal model, the increased light sensitivity and exaggerated tactile sensitivity by bright illumination seems to occur in both acute and chronic phases of PTH.

#### 2.2.3. Other Behaviors including Phonophobia, Anxiety and Depression

In addition to photophobia, PTH patients often experience phonophobia, the hypersensitivity to sounds. This symptom has been less addressed in the animal model, though Fanselow’s group reported phonophobia-like behavior using the fluid percussion model [39]. Animals after 2 days of injury showed increased freezing time on the exposure of white noise and also freezing behavior in the inter-trial period compared to the sham animals. Immunohistochemistry analysis suggested that the noise exposure activated lateral amygdala but not hippocampus, suggesting that the trait of phonophobia which is associated with PTH was evoked in the TBI animals by altering the brain regions involving fear memory, even though the relationship to other PTH symptoms such as cephalic allodynia was not examined [39]. Other psychological abnormalities, including post-traumatic stress disorder, depression and anxiety following TBI are the common comorbidities to PTH patients [7]. Accordingly, a substantial number of preclinical studies using the TBI models have been conducted to investigate the mental disorders. However, the anxiogenic and depressive behaviors in those preclinical studies have not been consistently demonstrated [40]. Nonetheless, it is helpful to determine whether there is a correlation between the PTH specific symptoms such as cutaneous allodynia and the psychological abnormalities. In the polytrauma injury model, male animals were found to travel a longer distance than the female animals in the open field test, indicating an anxiety-like behavior, although both male and female animals acquired cephalic allodynia [36]. Bree et al. reported that in the weight drop model, animals showed reduced rearing behavior as well as the cephalic tactile withdrawal threshold up to 1 week post-injury, but no differences were found in the novel object test and the open field test [29]. However, in their recent report, animals with a single weight drop injury showed not only rearing, but also duration time differences in the center zone in the open field test 3 days post-injury [18], suggesting that the TBI animals likely acquired an anxiety-like state. In the blast injury model, which showed cephalic hyperalgesia till 2 months post-injury, no significant differences were observed in the duration time in the center zone in the open field test, time in open arms in the elevated plus maze test and time in light zone in the light/dark box test [35]. In contrast, both anxiety-like behaviors observed in the elevated plus maze test and cephalic allodynia were sustained after 5 weeks in a cranium specific blast injury model, in which an injury device was used to selectively impact on the cranium but not the whole body in the awaken animals [34]. In contrast, there were no differences in the travel distance, time and entry number in the center zone examined by open field test in the weight drop model animals 8 days post-injury. There was also no significant difference in the adhesive removal test, although cephalic algesia was sustained up to 1 month post-injury [41]. Thus, the relationship between anxiety-like behavior and cutaneous allodynia has not been clarified in the animal model studies.

#### 2.2.4. Headache Inducing Agents in the PTH Models

To gain the mechanistic insight of PTH, several studies have been conducted on the use of headache-inducing agents in the PTH models. The commonly used headache triggers are nitric oxide donors which induce vascular dilation, such as nitroglycerin (NTG) or sodium nitroprusside (SNP). NTG administration generally causes transient hyperalgesia in animals [41] and human subjects [42]. Nitric oxide in the vascular endothelial cells activates soluble guanylyl cyclase to produce cGMP, which in turn activates signaling pathways dependent or independent upon the G-protein coupled receptors causing vasodilation [43]. Orofacial allodynia triggered by the vasodilator injection was significantly higher in the TBI than sham control animals. Even hyperalgesia induced by TBI was resolved after 2 weeks, administration of low dose of NTG exaggerated cephalic sensitivity in the injured animals on day 15 and day 30 post-injury [29], suggesting that NTG exploited a latent hyperalgesic state in the PTH animals. Moreover, it was reported that both cephalic and hind paw allodynia were induced by NTG 2 weeks post-injury when TBI induced allodynia was recovered [44]. Although high dose of NTG (10 mg/kg) did not differentiate between TBI and sham control, at low dose of NTG (0.1 mg/kg) only TBI animals showed hypersensitivity. These phenomena were consistently observed in the TBI animals at 4 weeks and 12 weeks post-injury. The subthreshold dose of SNP was also used to differentiate the orofacial and plantar tactile sensitivities between sham control and the repetitive TBI animals caused by weight drop. After TBI induced hyperalgesia was returned to the basal level 14 weeks post-injury, SNP (0.25 mg/kg) administration caused hypersensitivity in both cephalic and extracephalic regions in the TBI animals but not in the sham control animals [20], suggesting that the nitric oxide donors can trigger latent pain sensitization in the TBI animals.

Calcitonin gene-related peptide (CGRP) is known to be a vasodilating neuropeptide and plays a pivotal role in the migraine pathogenic mechanism. CGRP levels in plasma were found to increase during migraine attack [45], and intravenous infusion of CGRP causes migraine-like attacks in chronic migraineurs [46] as well as PTH patients [47,48]. In turn, the anti-CGRP medications including CGRP receptor antagonists and antibodies against CGRP and CGRP receptors have been used for the treatment of patients with primary migraine [49,50,51]. To examine whether CGRP affects nociceptive response in the PTH model, recombinant CGRP peptide was intraperitoneally injected to the weight drop TBI animals followed by von Frey test on days 7, 21, 35, and 91 post-injury. TBI animals with a low dose of CGRP (0.01 mg/kg) injection showed significantly higher cephalic hypersensitivity than sham animals until 91 days post-injury [20]. Thus, treatment with the headache inducing agents could provoke cephalic as well as extracephalic latent sensitivity in PTH animals when injury induced allodynia was no longer present. These results suggest that TBI animals are more sensitive to the pain-inducing vasodilating agents, which can be exploited using the subthreshold doses of these agents.

### 2.3. Pathologies of the Trigeminovascular System

#### 2.3.1. Trigeminal Ganglion Neurons and Glial Cells

The trigeminovascular system is critically involved in the initiation and propagation of PTH, although impairment of other brain regions after TBI also contributes to the PTH development. The trigeminovascular system innervates the cerebral arteries, dural and pial blood vessels and sinuses, where nociceptive sensitization is originated [52]. This system consists of three pseudounipolar sensory neurons in the trigeminal ganglion (TG), which give rise to the ophthalmic (V1), the maxillary (V2), and the mandibular (V3) branches. There is cross-activation among the three nerves within the TG and a close association between TG neurons and the surrounding satellite glial cells (SGCs). TG neurons are categorized based on the types of axon fibers. The Aδ fiber and Aβ fiber are myelinated by Schwann cells, whereas the small sized C fibers are unmyelinated. The TG axons centrally project to the trigeminal nucleus caudalis (TNC) located in the lateral medulla of the brain stem and extended to the C1 and C2 dorsal horn [53]. Once activated, TG neurons secrete several vasoactive neuropeptides such as CGRP, substance P, neurokinin A, and pituitary adenylate cyclase-activating polypeptide (PACAP) causing neurogenic inflammation. CGRP peptide biosynthesized in the TG neuronal soma is transported mainly through C fibers to the peripheral meninges and centrally to the brainstem. Conversely, the CGRP receptor components, such as the receptor activity modifying protein 1 (RAMP1) and calcitonin receptor–like receptor (CLR) are mainly localized in the Aδ fibers, in addition to glial cells [54,55].

Satellite glial cells (SGCs) are functionally similar to astrocytes, however, SGCs have a unique anatomical structure that surrounds the TG neurons. The close contact between SGCs and TG neurons implies an intimate cross-talk to modulate neuronal excitability and pain sensitivity [53]. Several reports indicated that SGCs contributed to nociceptive sensitization in the orofacial pain models [56,57]. One in vitro study showed that primary TG cells isolated from the orofacial pain model showed an increase in dye coupling and bidirectional electrical response between neuron and SGC and both were blocked by an inhibitor of the gap junction, indicating an important role of the gap junction on the cross-talk between neurons and SGCs under pathological conditions [58]. Knockdown of Connexin 43, a major gap junction component in SGC reduced pain-like behaviors in the animal model of chronic constriction injury of infraorbital nerve, suggesting that the gap junction mediated cross-talk can modulate neuronal sensitization [59]. Besides the gap junction mediated neuron-glia communication, Kir4.1, an inward rectifying potassium channel expressed in SGCs is shown to be responsible for controlling extracellular potassium ion to modulate TG neuronal activity [60], and its knockdown by dsRNA of Kir4.1 resulted in pain-like behaviors [61]. It is likely that suppression of Kir4.1 rectifying potassium current depolarizes SGCs, and induces the release of excitatory mediators to sensitize the nociception. These studies indicate that abnormal activation of SGCs contributes significantly to the facial pain. Neuroinflammation mediated by immune cells including SGC was reported in the TBI model [38]. iNOS immunoreactivity was observed in both neurons and non-neuronal cells, and was partially co-localized with CGRP in TG after 2 weeks of injury, but negligible in the control animals. Treatment with MK8825, a CGRP receptor antagonist, reduced the number of iNOS positive neurons. Consistently, previous in vitro study showed that CGRP treatment to primary SGC culture increased iNOS expression and nitric oxide release [62]. These studies suggest that the CGRP and nitric oxide signaling contributes to the pathogenesis of PTH.

#### 2.3.2. Meningeal and Cortical Regions

The meninges are three protective membrane layers encompassing the brain and spinal cord. They are composed of pia, arachnoid, and dura matters that contain blood vessels and enclose the cerebrospinal fluid. Studies from mild TBI patients and TBI animals demonstrated that brain trauma could cause meningeal vascular damage and cell death, as well as the breach of glial limitans [63]. Of note, CGRP biosynthesized in the TG is transported mainly through C fibers to the meningeal nerve endings. More CGRP was observed in the dura and pia maters in the CCI model compared to the sham control, [38]. The enhanced CGRP in meninges can activate the proximal CGRP receptor in the Aδ nerve terminals resulting in nociception [64,65].

Meningeal Mast cells (MCs) are localized in the vascularized tissues including skin and dura mater, especially near the blood vessels and in close apposition to the terminals of the dural afferents [66]. Meningeal MCs contain a substantial number of granules and secrete various inflammatory mediators, bio-organic amine (e.g., histamine and serotonin), cytokines, prostanoids, nitric oxide, and vasoactive neuropeptides in response to immunological activation [67]. The granules secreted by MCs in close proximity to sensory nerve endings can also modulate the excitability of nociceptors [68]. Therefore, MCs are localized in the strategic position to play a very important role in the immunological regulation in meninges, which in turn modulate trigeminal nociceptive sensitization. Levy’s group investigated MCs in the calvarial periosteum, the deep cranial tissue 48 h after weight drop concussive injury [69]. They found that the density of the periosteal MCs was not changed, but the degranulated MCs were significantly increased in the PTH animals. Moreover, when formalin as a nociceptive stimulator was given to the calvarial periosteum, PTH animals showed more frequent nociceptive behaviors such as scratching the head than the control animals, while no differences were found between PTH and control animals when formalin was applied to the hind paw. These results suggested that granule secretion from MCs in the periosteum plays a role in sensitization of trigeminal nerve nociception after closed head injury. The authors also performed a comparative study on the meningeal MC population and degranulation between the weight drop model and blast injury model [19]. In the weight drop model, MC degranulation reached a peak at an early time point (3 days post-injury) and then decreased, whereas in the blast model, MC degranulation continued for one month after injury. Despite both models showed significantly higher levels of MC degranulation at one month post-injury, the MCs density was not altered between the injured animals and the sham controls. Given that the weight drop model could generate focal axonal injury, the results suggested that MC degranulation was possibly affected by the direct concussive damage on meninges. In the blast model, MC degranulation was not changed primarily by the impact, but activation of MCs was increasingly sustained by diffuse axon injury and blood vessel damage over the time period. Recently they examined if degranulation of MCs modulates hypersensitivity of TG neurons [70]. When 48/80 compound which depletes MC granules was administered prior to injury, TBI induced allodynia was not altered by the drug treatment. However, at 29 days post-injury when TBI induced allodynia was resolved, reinstatement of cephalic allodynia by injection of NTG, a trigger of migraine in animal models, was blocked by 48/80 treatment. These results suggest that MCs granulation may not be critical for TBI induced allodynia in the acute phase, but might substantially contribute to the chronic sensitization.

Cortical spreading depression (CSD) is a pathophysiologic phenomenon characterized by transient cortical gray matter depolarization and subsequent hyperpolarization that propagate to the superficial cortical regions at a rate of 2 to 5 mm/min, resulting in suppression of spontaneous electrical activity [71]. CSD has been a characteristic in migraine patients with aura, but is also common in PTH patients irrespective of unpredictable occurrence and variance in duration and recurrence [72]. Induction of CSD in animals increased the CGRP levels [73], MC degranulation, and meningeal artery blood flow [74], and led to hypersensitization of trigeminal nociception [75]. Furthermore, activation of the second-order neurons was shown by increased firing rate [76], which can be the result of peripheral stimulation, as well as the central sensitization [77]. Resultantly, cephalic allodynia and anxiety-like behavior were elicited in the CSD models [78]. On the other hand, CSD was observed in several TBI models. For instance, Bouley et al. reported that TBI induced by a heavy but not a light weight drop developed CSD, and mice with CSD showed microbleeds in the injured hemisphere compared to animals without CSD [79]. Thus, monitoring CSD could be used as a biomarker for assessing the injury severity. It is likely that CSD plays an important role on the pathogenic mechanisms of PTH as shown in migraine with aura, but the extent to which CSD induced by TBI contributes to PTH remains to be determined.

#### 2.3.3. Brainstem Region

TG neurons innervate centrally to the TNC in the brainstem and C1 and C2 dorsal horn where the nociceptive signal is relayed to the second-order neurons, and then projects to thalamus and sensory cortex for processing the nociceptive signal. In turn, TNC neurons receive descending signals from several brainstem regions such as periaqueductal gray (PAG), locus coeruleus and rostral ventromedial medulla (RVM) as well as hypothalamus [80]. These multiple pathways of descending signals originating from the cortex are known to modulate peripheral nociceptive information in the pro- and anti-nociceptive manners. Lifshitz’s group investigated the neuropathology induced by TBI in brain areas related to the whisker somatosensory circuit. In the brainstem, the primary sensory information from the whisker is relayed to the principal trigeminal sensory nucleus, while whistler movement is transmitted via facial nucleus [81]. Microglial Iba1 immunoreactivity, which was localized near the swollen soma and thickened processes, was evidently increased in the principal trigeminal sensory nucleus 2 to 7 days after fluid percussion injury and lasted for 28 days, but the Iba1 immunoreactivity was not obvious in the facial motor nucleus. Silver staining revealed a significant neurodegeneration in the principal trigeminal nucleus but not in the facial nucleus till 7 days post-injury [82]. Thus, in the diffuse axonal injury model inflammatory neuropathology based on microglial activation and neurodegeneration was prominent in the trigeminal sensory nucleus rather than in the motor nucleus of the brainstem. In the controlled cortical impact induced closed head injury model, CGRP levels in the TNC were significantly increased by repeated injury until 1 week post-injury, which was consistent with the increased GFAP and Iba1 immunoreactivity [83]. In contrast, there were no significant changes in GFAP expression in the TNC 2 days after blast injury or Iba1 positive cells one month post-blast exposure, although anxiety-like behavior and cephalic allodynia were sustained for 3 to 5 weeks [34]. iNOS and CGRP were found to be co-localized in the TNC. Of note, based on the subcellular localization, iNOS seemed to be localized in the pre-synaptic afferent terminals of the TG neurons. iNOS knockout mice showed a reduction of CGRP levels 3 days post-injury, and the CGRP receptor antagonist MK8825 reduced iNOS expression [38]. These findings suggest that vasodilating nitric oxide production by iNOS and CGRP are interacted in the TG neurons. Elliot et al. reported that substance P was dramatically increased in the controlled cortical impact model 7 days post-injury and subsided till 28 days, and this profile was also seen with the Iba1 and GFAP expression [84]. On the other hand, the CGRP levels in the brainstem were increased by 3-fold and sustained for 28 days post-injury. The levels of CGRP and periorbital algesic thresholds were found inversely correlated, suggesting that CGRP in the brainstem plays an important role in cephalic hypersensitivity [84]. In 2 weeks following diffuse injury caused by weight drop, neurokinin 1R (NK1R), a receptor for substance P, GAD67, a key GABA biosynthetic enzyme, and serotonin were all significantly increased in the TNC. These results suggested that several neurogenic peptides and neurotransmission systems were activated in the TNC [33,85].

#### 2.3.4. Thalamus Region

Trigeminal nociceptive inputs from cranial vessels are relayed by the second-order TNC to the ventroposterior medial nucleus of thalamus (VPM), sensory cortex, and several subcortical areas such as reticular formation, cerebellum, and midbrain. Consistent with the results observed in TNC in the weight drop model, both serotonin and NK1R were significantly increased in the secondary somatosensory cortex and ventroposterior thalamus [33]. Conversely, in a blast injury model, there were no significant differences in GFAP immunoreactivity 2 days and 60 days post-injury in the posterior thalamus. Moreover, either spontaneous firing or neuronal response to the noxious facial stimulation in that region was not significantly different between TBI and control animals [34]. The GFAP or Iba1 immunoreactivity was also not changed in the VPM by blast injury [35]. On the other hand, Lifshitz’s group investigated the thalamocortical pathway related to whisker sensory circuit using the fluid percussion model, and found that activation of microglia and astrocytes in VPM was evident 7 days post-injury based on the morphological changes and the increased expression of inflammatory genes including translocator protein and GFAP [86,87]. In addition, extracellular glutamate levels and potassium-evoked glutamate release were increased in VPM [88]. Thalamus, particularly VPM is believed to be the site where the central processing and integration of trigeminal nociception is carried out [89]. Nociceptive information is further relayed to several different cortical regions including somatosensory cortex, motor cortex, anterior cingulate cortex, and prefrontal cortex through thalamocortical pathway [90]. Thus, there is no consensus for the pathological changes in the thalamus and its role in the development of PTH is still unclear.

**Figure 1:**
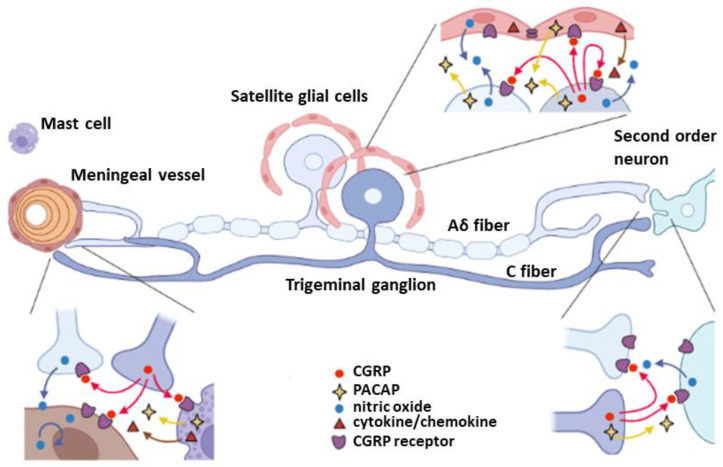
Schematic signal transduction pathways of TG neurons. There are mainly three modulatory regions that are important in nociceptive regulation of TG neurons; peripheral end terminal on the meningeal blood vessels, TG regions associating with satellite glial cells, and central terminal connecting to the second order neurons in TNC. Aδ fiber and C fiber neurons have distinct gene regulations, for instance CGRP and its receptors expression. CGRP plays a critical role in vasodilation as well as TG neuronal activity through induction of NO and cytokine/chemokine production, that can in turn trigger more CGRP release and nociception.

Figure 1 summarizes the intercellular communications of trigeminovascular system mediated by representative neuromodulators, nitric oxide, cytokine/chemokines, PACAP, and CGRP. The interactions with mast cells and vascular endothelial cells in meninges, satellite glial cells in TG, and second order neurons in TNC are shown in this schematic illustration. Other cells such as macrophage, microglia, and astrocytes that are not included in this diagram also contribute to the neuromodulation in the TG system.

## 3. Potential Therapeutic Drug Treatment on the PTH Models

According to the ClinicalTrials.gov, substantial number of PTH clinical trials have been planned, enrolled, or completed that include the use of CGRP monoclonal antibody (mAb), botulinum toxin, triptan, metoclopramide, prazosin, and others (https://clinicaltrials.gov/ct2/results?cond=posttraumatic+headache (accessed on 20 November 2022)). Current intervention for PTH is mostly based on the treatment strategy for primary headache because of its similar clinical presentations to migraine or tension-type headache. However, considering that the pathogenic mechanisms of PTH are likely to be distinct from that of a primary headache, development of the evidence-based PTH-specific treatment is desirable. Several therapeutic agents have been tested using the PTH models. The chief among them are CGRP related medication and sumatriptan, although other therapeutic substances including IL-2 [41,91], 48/80 secretagogue [70], oxytocin [31], SNC80, an delta opioid receptor agonist [44], and onabotulinum toxin A [92] were also reported (see Table 1). It is known that prostaglandins including PGE2 and PGI2 contribute to the nociceptive regulation in a migraine model [93], although the efficacy of NSAIDs in the treatment of PTH is uncertain. Given that modulation of the endocannabinoid system has been shown to be effective in the treatment of migraine and TBI, The potential therapeutic effect of endocannabinoid modulation in the treatment of PTH is also discussed.

### 3.1. Anti-CGRP Treatment

As mentioned above, the anti-CGRP treatment such as anti-CGRP mAb and CGRP receptor antagonists has been utilized in clinical interventions to migraine patients [95]. The therapeutic effect of a CGRP receptor antagonist, MK-8825 was examined in the controlled cortical impact TBI animal model [38]. At 2 weeks post-injury, administration of MK-8825 significantly alleviated cephalic allodynia and photophobia-like behavior. The expression of iNOS in the TNC and TG was reduced, suggesting that the production of nitric oxide and the subsequent oxidative stress were suppressed [38]. In a weight drop TBI rodent model, intraperitoneal administration of anti-CGRP mAb 2 h post-injury suppressed both cephalic and extracephalic allodynia examined at day 7 post-injury. Cutaneous allodynia induced by photophobic conditions on day 14 post-injury was significantly ameliorated when the CGRP mAb was administered at 2 h, day 7 and day 14 post-injury, but it was not effective when given only at day 10 post-injury [30]. Levy’s group reported that administration of the CGRP mAb to female TBI mice on days 0, 6, and 12 post-injury had a minimal effect on cephalic allodynia. However, repeated injection on day 0, 6, 13, 21, and 26 post-injury reduced the cephalic latent sensitivity evoked by NTG injection on day 30 post-injury [28]. The results suggested that the CGRP mAb did not exclusively ameliorate the allodynia in a short term, but was possibly effective for blocking the latent sensitization by external stimuli after TBI induced allodynia subsided. Recently, the therapeutic efficacy of CGRP mAb was examined based on the diffuse noxious inhibitory control (DNIC), the descending pain sensory circuit to negatively modulate pain sensation. When mice were stimulated by capsaicin on the forepaw as a conditioning stimulus, tail-flick latency as well as cephalic allodynia were increased till 60 min due to the inhibitory diffuse noxious control response governed by the central sensory system. In contrast, tail-flick latency and cutaneous allodynia were not inhibited by DNIC in the weight drop TBI animals. Under the DNIC test conditions, the CGRP mAb administration 2 h post-injury was found to reverse TBI induced cutaneous allodynia and also prevented the loss of DNIC [94]. These findings suggested that the CGRP signaling can modulate the descending pain modulatory circuit in the central nervous system.

### 3.2. Sumatriptan

Sumatriptan is currently used as a prescribed medication for acute migraine attack. Sumatriptan belongs to a chemical compound called triptan, the agonist of serotonin receptors especially 5-HT1B/5HT1D [96]. In addition to suppressing pathological vasodilation, sumatriptan was shown to reduce the serum CGRP levels of migraine patients [97] as well as to inhibit CGRP secretion from TG [98]. Blocking CGRP signaling is likely to be a major molecular mechanism of the anti-migraine effect of sumatriptan [99]. This drug has been tested in several PTH models to show significant therapeutic efficacy. It was reported that sumatriptan administration reversed periorbital allodynia induced by TBI and also reduced the pathological features such as the inflammatory markers in TG and the CGRP expression in TNC [38]. However, treatment with sumatriptan did not affect the dural MC degranulation induced by the weight drop injury [19]. For behavioral studies, cephalic response in the weight-drop model was alleviated by sumatriptan 3 days post-injury. Allodynia induced by NTG injection 14 days post-TBI was substantially reduced by sumatriptan treatment [29]. In a chronic migraine animal model induced by NTG injection every other day for 9 days followed by weight drop induced TBI, the NTG induced allodynia was significantly inhibited in both TBI and sham animals in the acute phase [44]. However, it was noticed that the tactile withdrawal threshold was progressively lowered by the repeated sumatriptan treatment in the sham control animals. Although the underlying mechanism is unclear, chronic treatment with sumatriptan could lead to an increase of nNOS and CGRP expressing TG neurons and therefore exacerbate the tactile sensitivity [29,100,101]. It is possible that the reduced tactile threshold was associated with medication overuse headache rather than PTH. In fact, cutaneous allodynia caused by chronic administration of sumatriptan was used for the model of medication overdose headache [100,101]. Thus, despite that the therapeutic effects of sumatriptan have been shown in the PTH model, the treatment regimens using sumatriptan should be carefully examined.

### 3.3. Endocannabinoid

The endocannabinoid (eCB) system is composed of amine or glycerol derivatives of N-acyl fatty acids such as 2-arachidonoylglycerol (2-AG) and N-arachidonoylethanolamide (AEA), the degrading and biosynthetic enzymes, and their binding receptors CB1, CB2, GRP55, and others [102]. It is well-known that eCBs as well as phytocannabinoids have potent anti-excitatory and anti-inflammatory properties and are protective in a variety of neurological diseases, although the underlying mechanisms remain to be determined [103]. In the last decade, several classes of inhibitors for the eCB degrading enzymes, Fatty acid amide hydrolase (FAAH), Monoacylglycerol lipase (MAGL), and others have been developed to enhance eCB efficacy to combat diseases [104]. Importantly, several eCBs, such as AEA, N-arachidonoyl GABA and N-arachidonoyl L-serine [105] were found to be the ligands not only for eCB receptors but also TRP ion channels that are molecular sensors to temperature, pH, pressure, and perception. These results indicate that eCBs play an important role in modulating inflammation and nociception [106]. To date, there are increased number of studies investigating the role of modulation of eCB system in the animal models of migraine or TBI [107], and several clinical trials have also been performed using synthetic cannabinoids and phytocannabinoids for migraineurs [108] and TBI patients [109].

#### 3.3.1. eCBs Modulation of the TG Activity In Vitro

It was reported that AEA administration to the TG culture increased miniature excitatory post-synaptic currents, and the increase was blocked by the TRP receptor antagonist capsazepine, but not the CB1 receptor antagonist AM251 [110], suggesting that the effect of AEA is mediated by TRP receptors. In addition, TG neurons were activated by AEA, N-arachidonyl dopamine, or arachidonyl-2-chlorethylamine to increase CGRP release, which was attenuated by the TRPV1 antagonists. Conversely, AEA or the cannabinoid receptor antagonists did not inhibit TRPV1 induced CGRP release, suggesting a mutual but complex interaction between TRPV1 and cannabinoid receptors in vitro [111]. In the primary TG neurons, AEA suppressed GABA evoked membrane current, which was reversed by AM251, but not capsazepine suggesting that eCBs can modulate TG neuronal activity through the CB1 receptor activation [112]. Consistently, the CB receptor agonist reduced GABAergic inhibitory postsynaptic currents in RVM neurons, which are known to modulate TNC activity [113]. AEA was also found to increase intracellular Ca^2+^ concentration in which adenylate cyclase and cGMP receptor were involved [114]. Recently, it was reported that MAGL enzymatic activity likely exceeded that of FAAH in spinal cord, brainstem, and TG based on the measurement by the activity-based protein profiling [115].

#### 3.3.2. eCBs in the Migraine Model

These results are consistent with the “clinical endocannabinoid deficiency” theory proposed by Russo based on the findings that eCBs tone was lower in painful conditions such as fibromyalgia, bowel syndrome, and migraine [116]. Previous investigation on the FAAH enzymatic activity as well as the eCBs levels including AEA in migraine patients showed inconsistent results partially due to the different migraine phenotypes (episodic, chronic or overuse medication) or biological fluids (CSF, plasma or blood) [117,118,119,120,121]. In the NTG induced migraine model, both the FAAH mediated hydrolysis activity and the eCBs binding sites were increased in mesencephalon and hypothalamus [122]. In the medication overuse headache model induced bv a prolonged injection of either sumatriptan or morphine, 2-AG levels were reduced in PAG but not in cortex, TG and TNC, whereas AEA was increased in cortex but not in PAG, TG, and TNC. Consistently, when DAGLα, the key enzyme of 2-AG biosynthesis, was pharmacologically inhibited, the levels of 2-AG but not AEA were reduced in PAG and visual cortex, as a result animals showed episodic headache-like behavior [123].

AEA administration prior to NTG injection reduced nociceptive behavior and attenuated neuronal activity in TNC based on the expression of c-fos [122,124]. The FAAH inhibitors URB597 and PF3845 were found to block NTG induced hyperalgesia through the CB1 receptor dependent mechanism [125]. Tassorelli’s group further investigated that URB937, which is a FAAH inhibitor effective in the peripheral but not centrally, showed anti-nociceptive effects in NTG-induced migraine as well as the control animals [126]. Consistently, c-fos expression in TNC was also suppressed [127]. The anti-nociceptive effect of URB937 in both acute and chronic migraine animal models was found to be dependent on activation of CB1 receptor and concomitantly the reduced expression of CGRP, substance P and cytokines in medulla and cervical spinal cord [126]. In line with these findings, femoral administration of AEA to rats was found to reduce the dural blood vessel dilation induced by CGRP, SNP or capsaicin. The inhibitory action of AEA was reversed by co-administration of the CB1 receptor antagonist [128]. It was recently reported that the CB receptor agonist methanandamide attenuated NTG-induced CGRP production and the enhancement of total and degranulated mast cells in dura [129]. Recently in an odor-induced migraine model in combination with restraint stress, orofacial and hindpaw hyperalgesia were attenuated by a grape seed extract dependent on CB1 and CB2 receptors [130].

#### 3.3.3. eCBs in the TBI Model

It was reported that eCB levels have been altered in several TBI animal models; in our previous study, AEA was showed to be increased in the ipsilateral cortex [131], which is consistent with a report by others [132]. In line with a previous report that elevated 2-AG levels in a closed head injury model [133], we also observed a moderate, but not a significant increase of 2-AG in a repetitive TBI mouse model [134]. Leishman et al. investigated the levels of eCBs and its related lipids using lipidomic approach in cerebellum, TG and TNC of the single and repetitive TBI rat models [135]. In that study, substantial numbers of lipid species including arachidonic acid and the prostaglandins were increased in the aforementioned regions. Remarkably, N-arachidonoyl glycine (NAGly) was found to be consistently increased in all the test conditions. Biosynthesis of NAGly is regulated by Peptidase M20 domain-containing 1, which is reportedly involved in the nociceptive behavior possibly through N-acyl amino acids metabolism [136]. Systemic injection of palmitoylethanolamide (PEA), an endogenous eCB-related fatty acid ethanolamide normalized psychiatric behaviors and inflammatory cytokine gene expression [137] and reduced lesion size [138] in the TBI mouse model, although it was not determined if those effects were mediated by CB receptors. Our group and others have demonstrated the therapeutic efficacies of inhibitors of eCB degrading enzymes, MAGL and FAAH in the TBI animal models. Augmentation of eCBs attenuates neuroinflammation, blood–brain barrier disruption, demyelination and neuronal death [134,139,140]. Electrophysiological properties of pyramidal neurons were altered to attenuate the neuronal hyperexcitability induced by TBI [141,142]. Some pathogenic protein species such as the aggregated form of TDP-43 and the phosphorylated tau, which may cause age-related neurodegenerative diseases and chronic traumatic encephalopathy were reduced [143]. Consequently, animal behavioral tests showed amelioration of motor and memory deficits [131,144]. The shift of cellular state of microglia and macrophages from a homeostatic to an anti-inflammatory phenotype induced by eCBs [145] may result in the attenuation of neuronal injury.

It is still speculated that the eCB signaling is effective for the treatment of PTH, although accumulating evidence indicates that exogenous cannabinoids and the inhibitors of the eCB degrading enzymes have potent therapeutic efficacy on TBI. Since eCBs interact not only with the CB receptors but also with the nociceptive receptors such as TRPV1 [106,146], the antinociceptive effects of eCBs to the neuropathic pain including PTH are likely more complicated compared to other pathological conditions. Therefore, it is necessary to further elucidate the underlying mechanisms of eCBs action and to identify the specific pain-associated eCB lipid species.

#### 3.3.4. Hypothetical Pain Modulation by eCBs in the PTH Model

**Figure 2:**
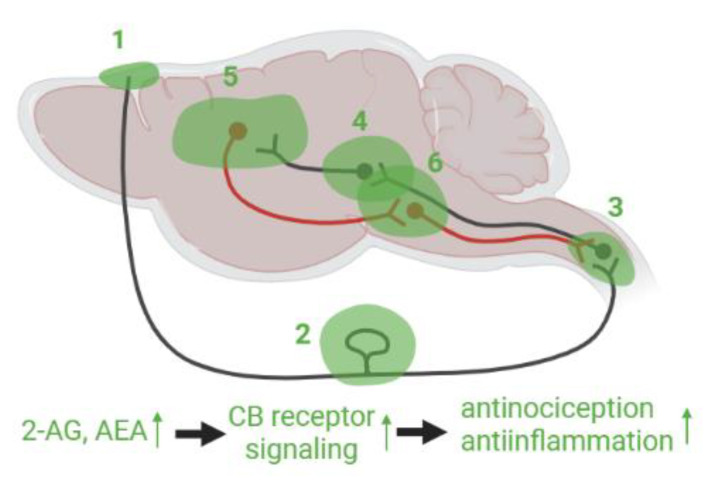
Hypothetical eCB mediated pain modulation in the PTH model. ECB signaling modulates nociceptive activation raised in TG neurons at (1) meninges by mast cells, macrophages and vascular cells, (2) TG with associated satellite glia cells, and (3) TNC. Nociceptive information ascends via the second order neurons innervating to (4) neurons in thalamus, amygdala, and other brainstem regions such as PAG where pain sensation was altered by CB receptor agonist or antagonist microinjection. Subsequently it is transited to (5) cortical regions such as mPFC and insular cortex. It is possible that eCB signaling has a substantial modulatory function, for instance through amygdala-mPFC-PAG pathway. In descending pathway, eCB system is deeply involved in the modulation via (6) PAG and RVM, and in (3) TNC. These brainstem regions integrate and process both ascending and descending pain signals via eCB system modulation.

Thus, it is promising that enhancement of eCBs by inhibition of the eCB catabolic enzymes or direct administration of eCB ligands can ameliorate PTH, because augmentation of eCB signaling has been demonstrated to mitigate migraine-like pathology and TBI induced neuronal damage. However, it is unknown how the eCB system can modulate the ascending and descending pain pathways in which eCB receptors and enzymes are substantially expressed. Based on previous findings including TBI, migraine, and other inflammatory and neuropathic pain models, we hypothesize that eCBs can modulate PTH in various regions (Figure 2). As mentioned above, the neuronal activity of TG neurons are regulated by CB1 receptor and eCBs. In addition to TG, mast cells activation such as degranulation is alleviated by a CB receptor agonist [129]. In the dural blood vessel, AEA caused vasodilation [147], in line with a fact that eCB degrading enzymes modulate the endothelium dependent vascular tone [148]. CGRP production is regulated in a CB1 receptor dependent manner in cervical spinal cord [126]. The increment of CGRP and substance P in orofacial pain model was reversed by the FAAH inhibitor in thalamus, hypothalamus, and mesencephalon [149], and microinjection of CB receptor agonist or antagonist modulated nociceptive response in brainstem, amygdala, and some nuclei in thalamus [150,151]. Among the pain regulating neural circuits, amygdala-mPFC pathway was well-addressed [152,153], since it is known that mPFC neuronal activity is attenuated by strong inhibitory signal from amygdala in the neuropathic pain model [154]. Basolateral amygdala pyramidal neurons project to GABAergic interneurons in mPFC, which predominantly express CB1 receptor. Under the pain state, GABAergic disinhibition was decreased since CB1 receptor expression was downregulated [153]. Therefore, mPFC pyramidal neuron activity was weakened, which leads to attenuation of the descending pain modulation. In addition, Neugebauer’s group reported that 2-AG tone in the mPFC pyramidal neurons was also lowered due to reduced mGluR5 mediated activation of 2-AG biosynthetic enzyme, DAGLα. Conversely, administration of agonists for mGluR5 and CB1 receptor restored GABAergic disinhibition and mPFC pyramidal neuron activity [152,155], which can further modulate the neuronal activity in PAG leading to descending inhibitory control of nociception [153]. Additionally, in the insular cortex, microinjection of the FAAH inhibitor attenuated hyperalgesia as well as neuronal activity in the region [156]. RVM and PAG are reciprocally connected modulating descending pain pathway in which the eCB signaling mediated by CB1 as well as CB2 receptor is deeply involved [157,158,159,160]. Microinjection of the CB agonists in PAG caused antinociceptive behavior [161] as well as reducing neuronal activity in RVM [162]. Conversely PAG neurons can modulate activity of RVM ON and OFF cells by injection of cannabidiol or FAAH inhibitor [162,163]. In fact, microinjection of CB1 agonist in the vlPAG attenuated the activity of trigeminal Aδ-fiber neurons that are activated by electric stimulation at the dura mater, suggesting that dural nociceptive response can be modulated from vlPAG through CB1 receptors [164].

## 4. Perspective

A recent study demonstrated that intravenous injection of CGRP in PTH patients exacerbated the migraine-like symptoms [47], suggesting that the CGRP plays an essential role in the PTH pathogenesis. Using the CGRP receptor blocker erenumab, one case series [165] and an open-label study [166] have been published recently. Similar to the migraine treatment, CGRP related medication seems to be a promising intervention for PTH, although the randomized clinical trials with placebo group are required to validate the therapeutic efficacy. Despite that the PTH symptoms are quite similar to those of the primary headache such as migraine- or tension-type headache, PTH has obviously distinct causes from primary migraine due to the brain injury. Indeed, by means of MRI, Schwedt’s group recently revealed the structural differences in some brain regions between PTH and migraine patients [167,168]. Of note, very few studies examined the CGRP levels in PTH patients, though one study showed that the plasma levels of CGRP is lower in persistent PTH than healthy control [169], which is contradictory to a notion that CGRP levels in plasma are increased in migraine patients [170]. In terms of the animal model, serum CGRP levels were increased 7 days post-injury especially in female rats [28]. The discrepancies in these studies may be caused by the different sampling protocol or the technical difficulties in the sample collection without a degradative loss [171]. There have been several studies showing the therapeutic effects of CGRP blockade in the PTH animal models [28,30,38,94], and one study demonstrated that low dose of CGRP injection caused hyperalgesia in a PTH model [20]. It is possible that the therapeutic effect on the latent sensitization may be more clinically relevant to the persistent PTH [28]. Accordingly, further investigations using animal models are needed to determine the CGRP dependent and independent mechanisms of PTH and to develop novel therapeutics for the prevention and treatment of PTH.

## Figures and Tables

**Table 1:** PTH models treated with potentially therapeutic drugs.

Species	Injurytype/Treatment	Pathology	Behavioral Change	Reference
ICR mice	weight drop		ceVF D1-11 ↓, hpVF D1-7 ↓	[92]
			ce&hpVF under lit D14, 28, 67 for 3–4 days ↓	
	onabotulinumtoxinA		2h PI reversed ce&hpVF D1–D67	
			72h PI reversed ceVF under lit D4, D14, D28	
			72h PI reversed hpVF under lit D4, D14	
			12D PI reversed ce&hpVF D14	
C57BL6 M	weight drop		ce&hpVF D1,D2 ↓	[94]
			DNIC D2 ↓	
	CGRP mAb 2hrPI		ce&hpVF reversed D1,D2	
			DNIC reversed D2	
SW mice	weight drop	TG CGRPR + cells,		[91]
		PACAP^+^cells ↑		
C57BL/6 M&F	IL-2	reversed by IL2		
		In CGRP KO, CGRPR^+^cells by NTG ND	CGRP KO, ceVF by NTG ND	
C56BL6	weight-drop		ceVF D1-28 ↓/reversed IL2	[41]
	IL2		OF time entry rearing D8 ND	
			rotarod D6 ND	
			adhesive test D12 ND	
	NTG D35-40		ceVF D39 ↓/reversed by IL2	
S-D rat F	weight drop	serum CGRP F ↑D7, not M	OF center time D2,3 ↓	[28]
			ceVF D2-21 ↓	
			hpVF ND	
	NTG D30		TBI ce&hpVF ↓	
			sham ce&hpVF ND	
	SUMA D7		ceVF reversed by SUMA	
	NTG + SUMA D30		ce&hpVF reversed by SUMA	
	CGRP mAb D0,6,12		ceVF ND by CGRP mAb	
	CGRP mAb D0,6,13,21,26 + NTGD30		ceVF reversed by CGRP mAb	
			hpVF ND by CGRP mAb	
S-D rat M	weight drop		OF rearing D3, 7, 14 ↓	[18]
	single heavy drop		OF center time D3 ↓	
			OF distance ND	
			ceVF D3–30 ↓	
			hpVF D14–42 ↓	
	GRP mAb every 6 day		ceVF D3,7,14,30 reversed by CGRP mAb	
			hpVF ND	
	repeat drop 72 h X3		OF rearing D3, 7, 14 ↓	
			OF center time ND	
			OF distance ND	
			ceVF ND	
			hpVF D14, D30 ↓	
C56BL6 M	weight-drop		ceVF D1-D10 ↓	[30]
	minimal anesthsia		hpVF D1-D10 ↓	
			Photo-stress followed ce&hpVF D14 ↓	
	CGRP mAb 2h PI		ce&hpVF reversed D1-D6	
	CGRP mAb D0,7,14PI		Photo-stressed ce&hpVF reversed D14	
	CGRP mAb D10PI		Photo-stressed ce&hpVF D14 ND	
S-D rat M	weight-drop	MC degranulation D7 ↑	ceVF D3,7,14 ↓	[70]
	CGRP mAb D0,6PI	MC degranulation ND		
	48/80 D-4PI	degranul MC↓/irregular MC↑	ceVF D3,7,14 ND by 48/80	
	NTG D29PI	degranulation D29 ↓	ce&hpVF D30 ↓	
	48/80 D-4	ND by 48/80	reversed by 48/80	
C57BL6J M	weight-drop	TG CGRP 2wk ↑	hpVF D3–D9 ↓	[44]
	NTG 2wkPI		hpVF NTG TBI ↓, sham ND	
	NTG 4wkPI		hpVF NTG TBI ↓, sham ND	
	NTG 12wkPI		hpVF NTG TBI ↓, sham ND	
	NTG + SUMA 2wkPI		hpVF TBI reversed by SUMA D3,5,7	
			hpVF sham SUMA ↓ D5,7,9	
	NTG + topiramate 2wkPI		hpVF TBI reversed by TOP D5,7,9	
			hpVF sham ND	
	NTG + SNC80 2wkPI		hpVF TBI reversed by SNC D5,7,9	
			hpVF sham ND	
S-D rat M	lateral fluid percussion		ce&hpVF D1–D35 ↓	[31]
	OXT intranasal		ce&hpVF reversed by OXT for 0.5–3 h	
	OXT + atosiban		ce&hpVF of OXT reversed by Atosiban for 0.5–4 h	
		intranasal injected OXT in TG↑	OXT preference test ↑	
S-D rat	weight drop		rearing behavior D2–D7 ↓	[29]
M			novel object recognition ND	
			thigmotaxis no change	
			ceVF D3, D7 ↓, hpVF ND	
	SUMA		ceVF reversed by SUMA	
	CGRP mAb		ceVF reversed by CGRP mAb	
	NTG D15, D30PI		ceVF D15, D30 TBI ↓, sham ND	
			hpVF D15 TBI ↓, sham ND	
			hpVF D30 TBI, sham ND	
	NTG + SUMA		ceVF D14 reversed by SUMA	
	NTG + CGRP mAb		ceVF D14 reversed by CGRP mAb	
			conditioned place aversion reversed CGRP mAb	
C57BL6	CCI	TNC&TG iNOS 2wk ↑	ceVF 2wk ↓	[38]
M		meningeal layer CGRP ↑	L/D box lit time 2wk ↓	
		TNC CGRP ↑	L/D box rearing in lit 2wk ↓	
	SUMA D13,14PI	TNC&TG iNOS reversed by SUMA	ceVF reversed by SUMA	
		TNC CGRP reversed by SUMA 2wk	L/D box lit entry ↓	
	MK8825 D13,14PI	TNC&TG iNOS reversed by MK	ceVF reversed by MK	
			L/D box lit time reversed by MK	
			L/D box rearing in lit reversed by MK	
iNOS KO		TNC CGRP reversed in KO D3	L/D box lit time reversed in KO	
			L/D box rearing in lit reversed in KO	

ceVF, cephalic von Frey test; F, female; hpVF, hind paw von Frey test; L/D box, Light/dark box; M, male; MK, MK8825; ND, not differentiated; OF, open field test; OXT, oxytocin; PI, post injury; S-D rat, Sprague Dawley rat; SUMA, sumatriptan; SW mice, Swiss Webster mice; wk, week.

## Abbreviations

2-AG, 2-arachidonoylglycerol; AEA, N-arachidonoylethanolamide; BSM, brainstem; CGRP, calcitonin gene-related peptide; CLR, calcitonin receptor–like receptor; CORT, corticotrophin; CRX, cortex; CSD, cortical spreading depression; DAGLα, diacylglycerol lipaseα; DNIC, diffuse noxious inhibitory control; eCB, endocannabinoid; EPM: elevated plus maze test; FAAH, fatty acid amide hydrolase; F, female; GABA, γ-aminobutyric acid; iNOS, inducible nitric oxide synthase; M, male; mAb, monoclonal antibody; MAGL, monoacylglycerol lipase; MC, mast cell; mTBI, mild traumatic brain injury; ND, not differentiated; NK1R, neurokinin 1R; NSAID, non-steroidal anti-inflammatory drug; NTG, nitroglycerin; OF: open field test; PAG, periaqueductal gray; PACAP, pituitary adenylate cyclase-activating polypeptide; PI, post injury; PO: posterior thalamus; Prv: principal trigeminal nucleus; PTH, posttraumatic headache; RAMP1, receptor activity–modifying protein 1; RGS, Rat Grimace Scale; S-D rat, Sprague Dawley rat; SGC, satellite glial cell; SNP, sodium nitroprusside; SP, substance P; TG, trigeminal ganglion; SW mice, Swiss Webster mice; TNC, trigeminal nucleus caudalis; TRPV1, transient receptor potential cation channel subfamily V member 1; VF, von Frey test; VIIN: facial motor nucleus; VPM, ventral posteriomedial thalamus.
